# Chronic obstructive pulmonary disease and periprocedural complications in patients undergoing percutaneous coronary interventions

**DOI:** 10.1371/journal.pone.0204257

**Published:** 2018-10-01

**Authors:** Rafał Januszek, Artur Dziewierz, Zbigniew Siudak, Tomasz Rakowski, Dariusz Dudek, Stanisław Bartuś

**Affiliations:** 1 2^nd^ Department of Cardiology and Cardiovascular Interventions, University Hospital, Krakow, Poland; 2 2^nd^ Department of Cardiology, Jagiellonian University Medical College, Krakow, Poland; 3 Faculty of Medicine and Health Sciences, Jan Kochanowski University, Kielce, Poland; 4 Department of Interventional Cardiology, Jagiellonian University Medical College, Krakow, Poland; San Giovanni Addolorata Hospital, ITALY

## Abstract

**Background:**

The relationship between chronic obstructive pulmonary disease (COPD) and periprocedural complications of percutaneous coronary interventions (PCIs) is influenced by several factors. We aimed to investigate the association between COPD, its complication type and rate in patients undergoing PCI.

**Methods:**

Data were prospectively collected using the Polish Cardiovascular Intervention Society national registry (ORPKI) on all PCIs performed in Poland between January 2015 and December 2016. COPD was present in 5,594 of the 221,187 patients undergoing PCI. We assessed the frequency and predictors of periprocedural complications in PCI.

**Results:**

Patients with COPD were elder individuals (70.3 ± 9.9 vs. 67 ± 10.8 years; p < 0.05). We noted 145 (2.6%) periprocedural complications in the COPD group and 4,121 (1.9%) in the non-COPD group (p < 0.001). The higher incidence of periprocedural complications in the COPD patients was mainly attributed to cardiac arrest (p = 0.001), myocardial infarctions (p = 0.002) and no-reflows (p < 0.001). COPD was not an independent predictor of all periprocedural complications. On the other hand, COPD was found to be an independent predictor of increased no-reflow risk (odds ratio [OR] 1.447, 95% CI 1.085–1.929; p = 0.01), and at the same time, of decreased risk of periprocedural allergic reactions (OR 0.117, 95% CI 0.016–0.837; p = 0.03).

**Conclusions:**

In conclusion, periprocedural complications of PCIs are more frequent in patients with COPD. COPD is an independent positive predictor of no-reflow and a negative predictor of periprocedural allergic reactions.

## Introduction

The prevalence of chronic obstructive pulmonary disease (COPD) in the general population is estimated at 7.6% [1]. Diagnosis of COPD increases the risk of cardiovascular diseases and resulting mortality [2]. 2.4–10% of patients undergoing percutaneous coronary intervention (PCI) have been diagnosed with COPD [3,4]. More importantly, it has been observed that patients with COPD treated with PCI have more comorbidities and a greater extent of coronary artery disease. In addition, COPD is associated with an increased risk of repeated revascularization after PCI [5]. Data availability on the incidence of periprocedural complications in patients undergoing PCIs is limited. It has been demonstrated that the incidence of periprocedural complications in patients undergoing PCIs has decreased in previous years and is estimated at 1–3%. However, other studies have shown higher frequencies [6]. Several predictors of periprocedural complications, including advanced age, coronary plaque burden, chronic total occlusions (CTOs), coronary artery tortuosity, gender, PCI overall volume at one center and daily PCI volume have been demonstrated [7,8]. There are limited and conflicting data regarding periprocedural complications of PCI in patients with COPD. Some publications demonstrated that the overall complication rate was significantly lower in those with COPD. Whereas, patients with COPD had significantly higher incidences of death, major entry site complications and longer length of hospital stay [5].

The aim of this study was to assess the relationship between COPD, periprocedural complications and their predictors in patients treated with PCI.

## Materials and methods

### Study population, design and definitions

We analyzed prospectively collected national data from all patients who underwent PCIs in Poland between January 2015 and December 2016. Data on PCI practice in Poland were obtained from the ORPKI Polish National dataset which is coordinated nationwide by Jagiellonian University Medical College in cooperation with AISN PTK (Association of Cardiovascular Interventions–The Polish Cardiac Society). The method of collecting data in the ORPKI registry was presented in previously published works [9,10]. Consecutive patients with COPD were included. COPD was defined on the basis of previously established diagnosis. This fact was taken into account on the basis of existing medical records, including discharge cards and the typical treatment used for COPD. All indices recorded in the ORPKI database are based on periprocedural data uploaded by the operator after each procedure. Therefore, they do not include all in-hospital complications, mainly those which occurred after the procedure until discharge from the hospital. Also, we did not collect follow-up data after discharge. The diagnosis of all other periprocedural complications including death, cardiac arrest, puncture site bleeding, no-reflow, cerebral stroke, coronary artery perforation (CAP), coronary artery dissection, allergic reactions ultimately depended on the operators’ decisions. Periprocedural major adverse cardiac events (MACCE) were defined as the combination of all-cause deaths, myocardial infarction (MI) and cerebral stroke. The thrombolysis in myocardial infarction (TIMI) grade flow was used to estimate procedural angiographic effectiveness. PCI was considered effective when TIMI grade 3 was obtained after the procedure. The overall complication rate was presented as the number of patients with defined periprocedural complications, even if the particular patient presented more than one complication.

### Statistical analysis

All continuous variables were evaluated with the Kolmogorov-Smirnov test for distribution. Continuous variables are presented as mean ± standard deviation and median ± interquartile range. Categorical variables are presented as numeric values and percentages. Continuous variables were compared using the two-tailed Student’s *t*-test and the Mann-Whitney U-test, whereas categorical variables via the *χ*^*2*^ test. ANOVA was used to compare data between following years. To identify predictors of all periprocedural complications and those more specific such as allergic reactions and no-reflow phenomenon in the overall group of patients undergoing PCI, univariate and multivariate analyses were performed. Both, univariate and multivariate regression models for MACCE were constructed. A model based on the retrograde correction method was created. In this analysis, the following variables were tested: age, gender, diabetes, previous cerebral stroke, MI, PCI, coronary artery by-pass grafting (CABG), smoking status, psoriasis, kidney disease, COPD, vascular access, fractional flow reserve, intravascular ultrasound, optical coherence tomography, thrombectomy, rotablation (RA), pharmacological treatment, baseline TIMI flow, contrast and radiation dose, gender, clinical presentation of coronary artery disease (CAD), type of coronary artery lesions including distribution, bifurcations and chronic total occlusion (CTO) procedures. A *p* value lower than 0.05 was considered to be significant. The statistical analyses were performed using Statistica 10.0 software (Dell Software, Inc, Round Rock, TX, USA).

## Results

**Fig 1:**
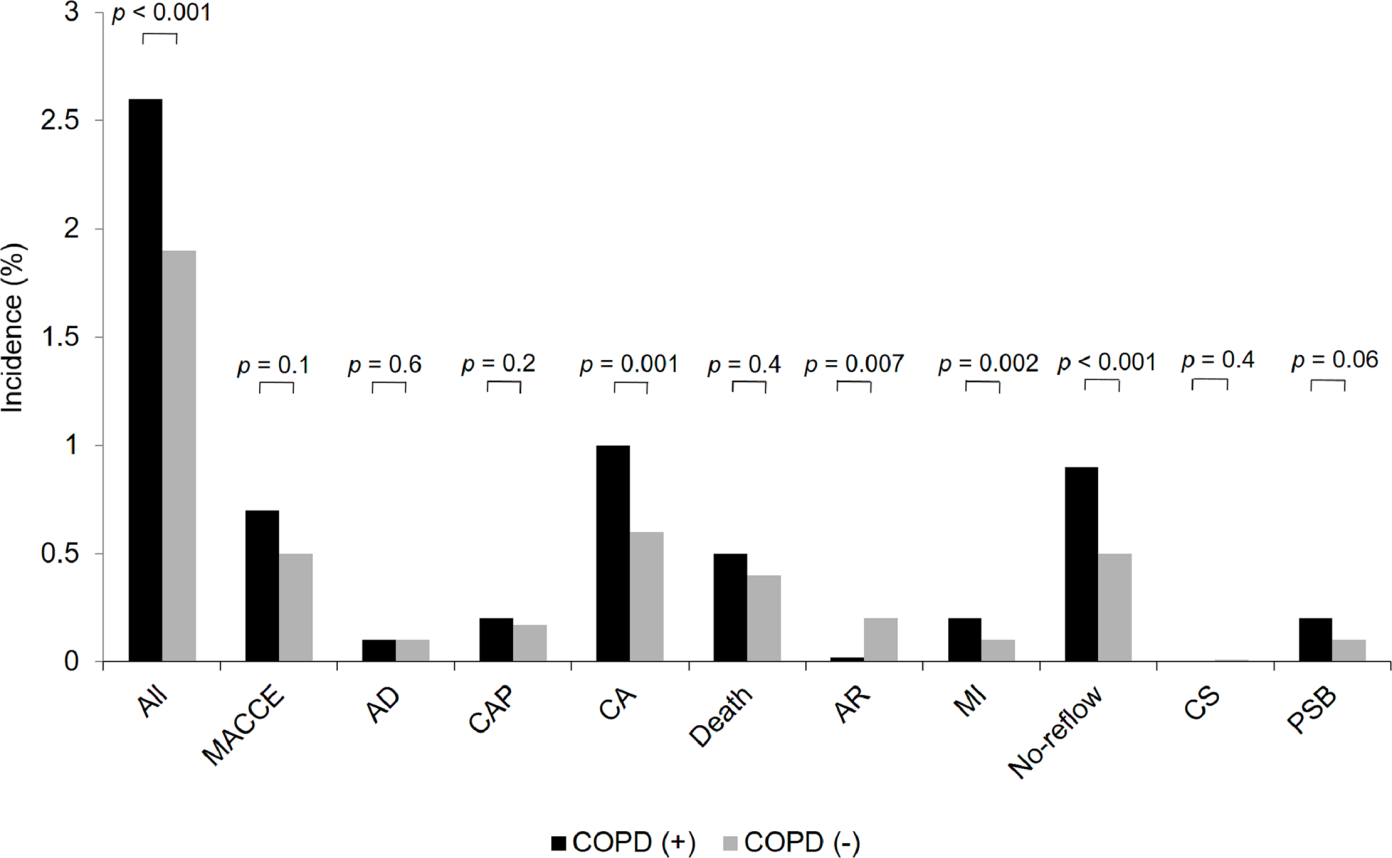
The incidence of periprocedural complications in the COPD and non-COPD groups. **Data are presented as % unless otherwise indicated.** Abbreviations: AD, arterial dissection; AR, allergic reactions; CA, cardiac arrest; CAP, coronary artery perforation; CS, cerebral stroke; MACCE, major adverse coronary and cerebrovascular events; MI, myocardial infarction; NS, not significant; PSB, puncture site bleeding.

We analyzed 221,187 PCIs performed in Poland between January 2015 and December 2016. Among all PCI patients, 5,594 had COPD (2.5%). We found 145 overall periprocedural complications in 5,594 patients with COPD (2.6%) and 4,121 in 215,593 patients from the non-COPD group (1.9%) (p < 0.001) (Fig 1). It was mostly determined by the greater percentage of cardiac arrests (1% vs. 0.6%; p = 0.001), MIs (0.2% vs. 0.1%; p = 0.002), and no-reflows (0.9% vs. 0.5%; p < 0.001) in the COPD group as compared to the non-COPD group (Fig 1). Periprocedural allergic reactions were less frequent in the COPD group (0.02% vs. 0.2%; p = 0.007). Other periprocedural complications did not differ significantly between both groups (Fig 1).

### Periprocedural complications and clinical presentation of CAD

ST-segment elevation MI (STEMI) occurred in 994 patients (17.7%), while non-ST-segment elevation MI (NSTEMI) took place in 1,384 patients (24.7%). More complications occurred in STEMI subgroup compared to NSTEMI, which was driven by a higher rate of cardiac arrests (p = 0.007), deaths (p = 0.02) and no-reflows (p < 0.001) (Table 1). The angiographic success was significantly lower in STEMI as compared to NSTEMI (p = 0.001). The usage rate of thrombectomy was higher in the STEMI group (p < 0.001) (Table 1). There were no significant differences in vascular access between those two groups. Differences in the location of culprit lesion in STEMI and NSTEMI patients is presented in Table 1. Stable angina (SA) occurred in 1,549 patients, while acute coronary syndrome (ACS) was noted in 3,980 individuals (72%). Among patients from the COPD group, complication rate was higher in patients with ACS compared to SA (1.2% vs. 3.1%; p < 0.001), with the most significant contribution of cardiac arrest (0.6% vs. 1.2%; p = 0.005), death (0% vs. 0.7%; p < 0.001) and no-reflow (0.5% vs. 1.1%; p = 0.04) (Table 1). This relationship was present despite the fact that PCI of CTOs (p = 0.01), bifurcations (p = 0.01) and RAs (p < 0.001) were more common in SA patients. The angiographic success expressed as TIMI grade 3 flow was higher in the SA group (p < 0.001). Femoral access (FA) was more common in STEMI patients (p = 0.02) (Table 1).

**Table 1:** Selected indices in COPD group according to clinical presentation of CAD (NSTEMI vs. STEMI) before PCI and SA vs. ACS.

	COPD (+), n = 5,594 (100)		P-value	COPD (+), n = 5,529 (100)		P-value
	STEMI n = 994 (17.7)	NSTEMI n = 1,384 (24.7)		SA n = 1,549 (28)	ACS n = 3,980 (72)	
Age, years	70.3 ± 10.0 70 [63 ÷ 78]	71.4 ± 9.6 71 [64 ÷ 79]	0.006	69.5 ± 8.6 69 [63 ÷ 76]	70.7 ± 9.5 70 [64 ÷ 78]	<0.001
All complications	62 (6.2)	38 (2.7)	<0.001	19 (1.2)	122 (3.1)	<0.001
Arterial dissection	1 (0.1)	3 (0.2)	0.55	0 (0)	5 (0.1)	0.1
CAP	3 (0.3)	3 (0.2)	0.68	4 (0.2)	10 (0.2)	0.9
Cardiac arrest	26 (2.6)	16 (1.1)	0.007	6 (0.6)	48 (1.2)	0.005
Death	17 (1.7)	10 (0.7)	0.02	0 (0)	29 (0.7)	<0.001
Allergic reactions	0 (0)	0 (0)	-	0 (0)	1 (0.02)	0.5
No-reflow	28 (2.8)	9 (0.6)	<0.001	8 (0.5)	43 (1.1)	0.04
Cerebral stroke	0 (0)	0 (0)	-	0 (0)	0 (0)	-
Puncture site bleeding	4 (0.4)	3 (0.2)	0.4	1 (0.06)	9 (0.2)	0.2
Chronic total occlusion	38 (3.8)	55 (4.0)	0.85	91 (5.9)	171 (4.3)	0.01
Bifurcation	51 (5.1)	91 (6.6)	0.14	122 (7.9)	244 (6.1)	0.01
Rotablation	1 (0.1)	4 (0.3)	0.32	18 (1.2)	11 (0.3)	<0.001
Thrombectomy	109 (10.9)	27 (1.2)	<0.001	-	141 (3.5)	-
TIMI 3 after PCI	841 (88.1)	1,237 (92.2)	0.001	1,438 (92.8)	3,529 (91.8)	0.2
Location of culprit lesion						
Left main coronary artery	41 (4.1)	95 (6.9)	0.004	54 (3.5)	206 (5.2)	0.007
Left anterior descending	372 (37.4)	475 (34.3)	0.11	512 (33.0)	1,385 (34.8)	0.2
Circumflex branch	145 (14.6)	379 (27.4)	<0.001	391 (25.2)	890 (22.4)	0.02
Intermediate branch	17 (1.7)	20 (1.4)	0.6	25 (1.6)	52 (1.3)	0.38
Right coronary artery	377 (37.9)	380 (27.4)	<0.001	483 (31.2)	1,256 (31.5)	0.7
Saphenous vein graft	9 (0.9)	23 (1.7)	0.11	14 (0.9)	54 (1.3)	0.2
Vascular access						
Right radial artery	584 (58.7)	820 (59.2)	0.8	941 (60.7)	2,343 (58.9)	0.2
Left radial artery	139 (14.0)	220 (15.9)	0.19	250 (16.1)	611 (15.3)	0.5
Femoral artery	264 (26.5)	344 (24.8)	0.34	340 (21.9)	987 (24.8)	0.02
Others	7 (0.7)	18 (1.3)	0.15	18 (1.2)	39 (1.0)	0.5

Data are presented as % unless otherwise indicated.

Abbreviations: ACS, acute coronary syndrome; CAD, coronary artery disease; CAP, coronary artery perforation; NSTEMI, non-ST segment elevation myocardial infarction; SA, stable angina; STEMI, ST-segment elevation myocardial infarction; TIMI, thrombolysis in myocardial infarction.

### Periprocedural complications and distribution of coronary artery atherosclerosis

The COPD group consisted of 3,381 patients with single-vessel disease (SVD) (65.6%) and 1,771 individuals with multi-vessel disease (MVD) +/- left main coronary artery (LMCA) involvement and isolated LMCA disease (34.4%). The rate of MVD patients with LMCA involvement (p = 0.02), without LMCA involvement (p < 0.001) and isolated LMCA disease (p < 0.001) was significantly higher in the COPD group compared to the non-COPD group. Complication rate was almost two times higher in the MVD group compared to the SVD group (p < 0.001), which was mainly due to higher rate of MACCEs (p < 0.001), dissections (p = 0.01), cardiac arrests (p < 0.001), deaths (p < 0.001) and no-reflows (p = 0.04) (Table 2). In the MVD group, there were twice as many PCIs of bifurcations (p < 0.001), many more occurrences of LMCA PCIs (p < 0.001), significantly more PCIs of LAD (p < 0.001) and Cx (p < 0.001), while there were less PCIs of RCA (p < 0.001). We did not confirm any relationship between particular complications and location of the culprit lesion in specific coronary arteries. In the MVD group, there were significantly more patients with NSTEMI (p < 0.001) and STEMI (p = 0.02) presentation of CAD, while less with SA (p < 0.001). Differences in vascular access are presented in Table 2.

**Table 2:** Selected indices in the COPD group according to the distribution of coronary artery atherosclerosis and TIMI flow grade before PCI.

	COPD (+), n = 5,152 (100)		P-value	COPD (+), n = 5,392 (100) TIMI flow before PCI		P-value
	Non-SVD n = 1,771 (34.4)	SVD n = 3,381 (65.6)		0–1 n = 1,632 (30.3)	2–3 n = 3,760 (69.7)	
Age, years	71.2 ± 9.5 71 [64 ÷ 77]	69.8 ± 9.2 69 [63 ÷ 77]	<0.001	70.2 ± 9.9 70 [63 ÷ 78]	70.4 ± 8.9 70 [64 ÷ 77]	0.35
All complications	69 (3.9)	68 (2.0)	<0.001	76 (4.6)	65 (1.7)	<0.001
Arterial dissection	5 (0.3)	1 (0.03)	0.01	3 (0.2)	3 (0.1)	0.37
CAP	3 (0.2)	9 (0.3)	0.49	4 (0.2)	10 (0.3)	0.89
Cardiac arrest	30 (1.7)	21 (0.6)	<0.001	34 (2.1)	19 (0.5)	<0.001
Death	20 (1.1)	9 (0.3)	<0.001	22 (1.3)	7 (0.2)	<0.001
Allergic reactions	0 (0)	1 (0.03)	0.46	0 (0)	1 (0.03)	0.51
Myocardial infarction	6 (0.3)	4 (0.1)	0.08	6 (0.3)	6 (0.2)	0.13
No-reflow	24 (1.3)	26 (0.8)	0.04	33 (2.0)	19 (0.5)	<0.001
Cerebral stroke	0 (0)	0 (0)	-	0 (0)	0 (0)	-
Puncture site bleeding	5 (0.3)	5 (0.1)	0.29	3 (0.2)	5 (0.1)	0.65
Chronic total occlusion	86 (4.8)	151 (4.4)	0.52	7 (0.4)	82 (2.2)	<0.001
Bifurcation	181 (10.2)	158 (4.7)	<0.001	126 (7.7)	239 (6.3)	0.06
Rotablation	9 (0.5)	18 (0.5)	0.9	6 (0.4)	20 (0.5)	0.42
Thrombectomy	56 (3.2)	87 (2.6)	0.22	114 (7.0)	23 (0.6)	<0.001
TIMI 3 after PCI	1,599 (92.3)	3,012 (92.4)	0.89	1,327 (81.6)	3,653 (97.4)	<0.001
Location of culprit lesion						
LMCA	209 (11.8)	6 (0.2)	<0.001	53 (3.2)	204 (5.4)	<0.001
Left anterior descent	675 (38.1)	1,097 (32.4)	<0.001	504 (30.9)	1,334 (35.5)	0.001
Circumflex branch	507 (28.6)	658 (19.5)	<0.001	357 (21.9)	903 (24.0)	0.08
Intermediate branch	31 (1.7)	39 (1.1)	0.07	18 (1.1)	55 (1.5)	0.29
Right coronary artery	479 (27.0)	1,187 (35.1)	<0.001	578 (35.4)	1,129 (30.0)	<0.001
Saphenous vein graft	24 (1.3)	42 (1.2)	0.73	22 (1.3)	46 (1.2)	0.7
Vascular access						
Right radial artery	1,023 (57.7)	2,048 (60.6)	0.05	957 (58.6)	2,251 (59.8)	0.39
Left radial artery	266 (15.0)	529 (15.6)	0.55	249 (15.2)	583 (15.5)	0.81
Femoral artery	461 (26.0)	772 (22.8)	0.01	411 (25.2)	886 (23.6)	0.2
Others	21 (1.2)	32 (0.9)	0.4	15 (0.9)	40 (1.1)	0.62
Clinical presentation						
Stable angina	370 (20.9)	1,047 (31.0)	<0.001	246 (15.1)	1,243 (33.1)	<0.001
Unstable angina	522 (29.5)	1,041 (30.8)	0.32	326 (20.0)	1,223 (32.6)	<0.001
NSTEMI	547 (30.9)	742 (21.9)	<0.001	433 (26.5)	906 (24.1)	0.059
STEMI	310 (17.5)	512 (15.1)	0.02	604 (37.0)	346 (9.2)	<0.001
Others	21 (1.2)	37 (1.1)	0.76	22 (1.3)	36 (0.9)	0.2

Data are presented as % unless otherwise indicated.

Abbreviations: CAP, coronary artery perforation; LMCA, left main coronary artery; MVD, multi-vessel disease; NSTEMI, non-ST segment elevation myocardial infarction; PCI, percutaneous coronary intervention; SVD, single-vessel disease; STEMI, ST-segment elevation myocardial infarction; TIMI, thrombolysis in myocardial infarction.

### Gender and periprocedural complications

There were 4,045 males (72.3%) and 1,547 females (27.7%) in the COPD group (Table 3). All of the complications more frequently occurred in females compared to the males (2.3% vs. 3.3%, p = 0.04). This was mainly due to higher incidence of MI (0.1% vs. 0.4%, p = 0.01), which occurred despite the fact that more PCIs of bifurcations (p < 0.001) and CTOs (p < 0.001) were performed in males (Table 3). It seems, that the higher prevalence of complications in women can be mainly owed to the higher STEMI (p < 0.001) and lower SA (p < 0.001) incidences in individuals before PCI which resulted in the more frequent use of thrombectomy (p = 0.01) (Table 3).

**Table 3:** Selected indices in the COPD group according to gender before PCI.

Variable	COPD (+), n = 5,592 (100)		P-value
	Males n = 4,045 (72.3)	Females n = 1,547 (27.7)	
Age, years	69.8 ± 9.3 69 [63 ÷ 77]	71.8 ± 9.1 72 [65 ÷ 79]	<0.001
All complications	94 (2.3)	51 (3.3)	0.04
Arterial dissection	3 (0.09)	3 (0.2)	0.24
CAP	11 (0.3)	3 (0.2)	0.6
Cardiac arrest	35 (0.9)	20 (1.3)	0.14
Death	19 (0.5)	11 (0.7)	0.26
Allergic reactions	0 (0)	1 (0.06)	0.1
Myocardial infarction	5 (0.1)	7 (0.4)	0.01
No-reflow	36 (0.9)	16 (1.0)	0.61
Cerebral stroke	0 (0)	0 (0)	-
Puncture site bleeding	6 (0.1)	4 (0.2)	0.38
Chronic total occlusion	215 (5.3)	52 (3.4)	0.002
Bifurcation	292 (7.2)	77 (5.0)	0.002
Rotablation	22 (0.5)	7 (0.4)	0.67
Thrombectomy	91 (2.2)	52 (3.4)	0.01
TIMI effectiveness (0-2/3)	3,639 (94.0)	1,384 (92.6)	0.9
Location of culprit lesion			
Left main coronary artery	203 (5.0)	60 (3.9)	0.7
Left anterior descent	1,389 (34.3)	528 (34.1)	0.88
Circumflex branch	939 (23.2)	355 (22.9)	0.83
Intermediate branch	60 (1.5)	18 (1.2)	0.36
Right coronary artery	1,234 (30.5)	533 (34.4)	0.004
Saphenous vein graft	56 (1.4)	12 (0.7)	0.06
Vascular access			
Right radial artery	2,408 (59.5)	913 (59.0)	0.72
Left radial artery	606 (15.0)	263 (17.0)	0.06
Femoral artery	988 (24.4)	356 (23.0)	0.26
Other	43 (1.1)	15 (1.0)	0.75
Clinical presentation			
Stable angina	1,176 (29.1)	373 (24.1)	<0.001
Unstable angina	1,151 (28.5)	450 (29.1)	0.63
NSTEMI	983 (24.3)	400 (25.9)	0.52
STEMI	686 (17.0)	308 (19.9)	0.009
Others	44 (1.1)	14 (0.9)	0.54

Data are presented as % unless otherwise indicated.

Abbreviations: CAP, coronary artery perforation; NSTEMI, non-ST-segment elevation myocardial infarction; STEMI, ST-segment elevation myocardial infarction; TIMI, thrombolysis in myocardial infarction.

### Vascular access and periprocedural complications

FA was used in 1,344 individuals (24%), while radial access was implemented in 4,250 patients (76%). A trend towards a higher risk of periprocedural complications in patients with FA was observed (3.2% vs. 2.4%; p = 0.1). Similarly, when considering each complication separately, there were no significant differences. However, we have noticed discrepancies in several other features. Patients from the FA group were older (71.1 ± 8.9 years vs. 70.1 ± 9.3 years; p < 0.001) and underwent more PCIs with RA (1.0% vs. 0.3%; p = 0.002). They presented STEMI before PCI more often (19.6% vs. 10.1%; p = 0.03), while undergoing thrombectomy less often (1.7% vs. 2.8%, p = 0.02). They also presented SA before PCI less frequently (25.3% vs. 28.5%; p = 0.02). There were no differences in location of culprit lesion between femoral and radial access subgroups and angiographic success. No differences were found between PCI of CTOs and bifurcation rate as well.

### Pre-PCI TIMI flow and periprocedural complications

Limited blood flow (TIMI 0–1) was found in 1,632 patients (30.3%), while preserved blood flow was noted in 3,760 individuals (69.7%) before PCI. All complication rates were higher in the TIMI 0–1 group compared to the TIMI 2–3 group assessed before PCI (p < 0.001). This was mostly caused by higher percentages of MACCE (p < 0.001), cardiac arrests (p < 0.001), deaths (p < 0.001) and no-reflows (p < 0.001) (Table 2). The number of patients with STEMI (p < 0.001) was greater in the group of patients with limited blood flow, whereas there were fewer patients with SA and UA (p < 0.001). The higher rate of complications in patients with the TIMI 0–1 grade flow seems to be mostly attributed to the higher rate of STEMI, despite the fact that there were more patients undergoing PCI of CTOs (*p* < 0.001) and LMCA (p < 0.001) in patients with preserved blood flow before PCI (Table 2).

### Comparing 2015 with 2016

We observed an increase in the number of patients with COPD (2.4% vs. 2.6%; p = 0.006). In 2016, there were more patients with SA (23.9% vs. 31.3%; p < 0.001) but less with STEMI (26.3% vs. 23.3%; p = 0.009) and NSTEMI (21.3% vs. 14.6%; p < 0.001). The FA approach was more common in 2016 (57% vs. 61.5%; p < 0.001), whereas RA was less common (25.4% vs. 22.8%; p = 0.02). In 2016, we noticed an increase in complexity of PCIs in the COPD group. The rate of PCIs within bifurcations (3.3% vs. 9.6%; p < 0.001) and CTOs (3.3% vs. 6.1%; p < 0.001) increased as compared to 2015. Considering the type of implanted stents, in 2016 we noted an increased percentage of DES stents (79.4% vs. 87.0%; p < 0.001) and a decrease in BMS (8.1% vs. 2.6%; p < 0.001) and BVS stents (1.6% vs. 0.4%; p < 0.001). Radiation exposition and contrast dose also decreased significantly in 2016 compared to 2015 (p < 0.001).Considering these changes, the overall incidence of all complications in the COPD group decreased in 2016 as compared to 2015, but without statistical significance (2.9% vs. 2.3%; p = 0.15).

### Predictors of all periprocedural complications, allergic reactions and no-reflows

**Fig 2:**
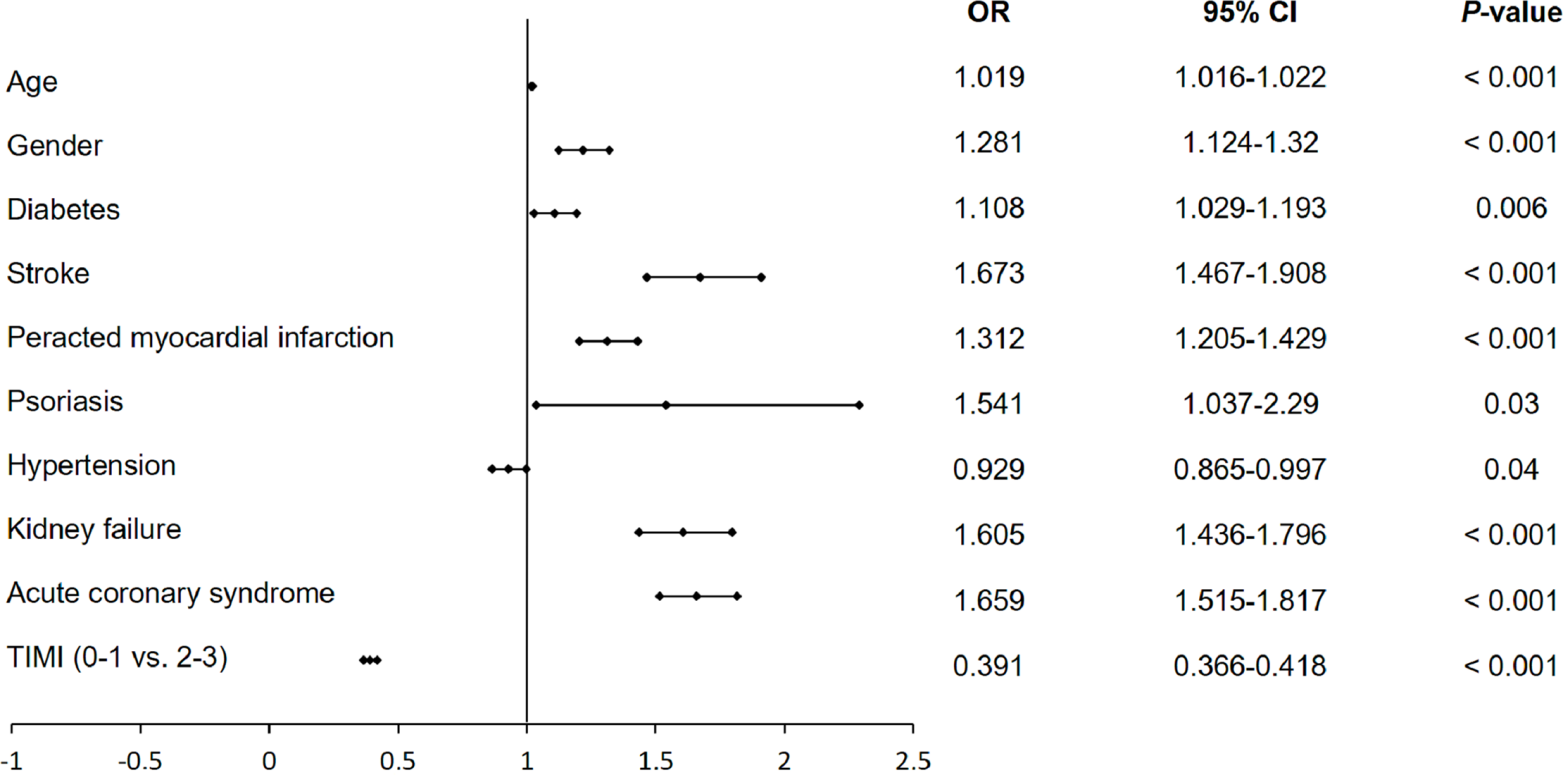
Predictors of all periprocedural complications assessed by multivariate analysis in the overall group of patients undergoing PCI. Abbreviations: CI, confidence interval; OR, odds ratio; TIMI, thrombolysis in myocardial infarction.

**Fig 3:**
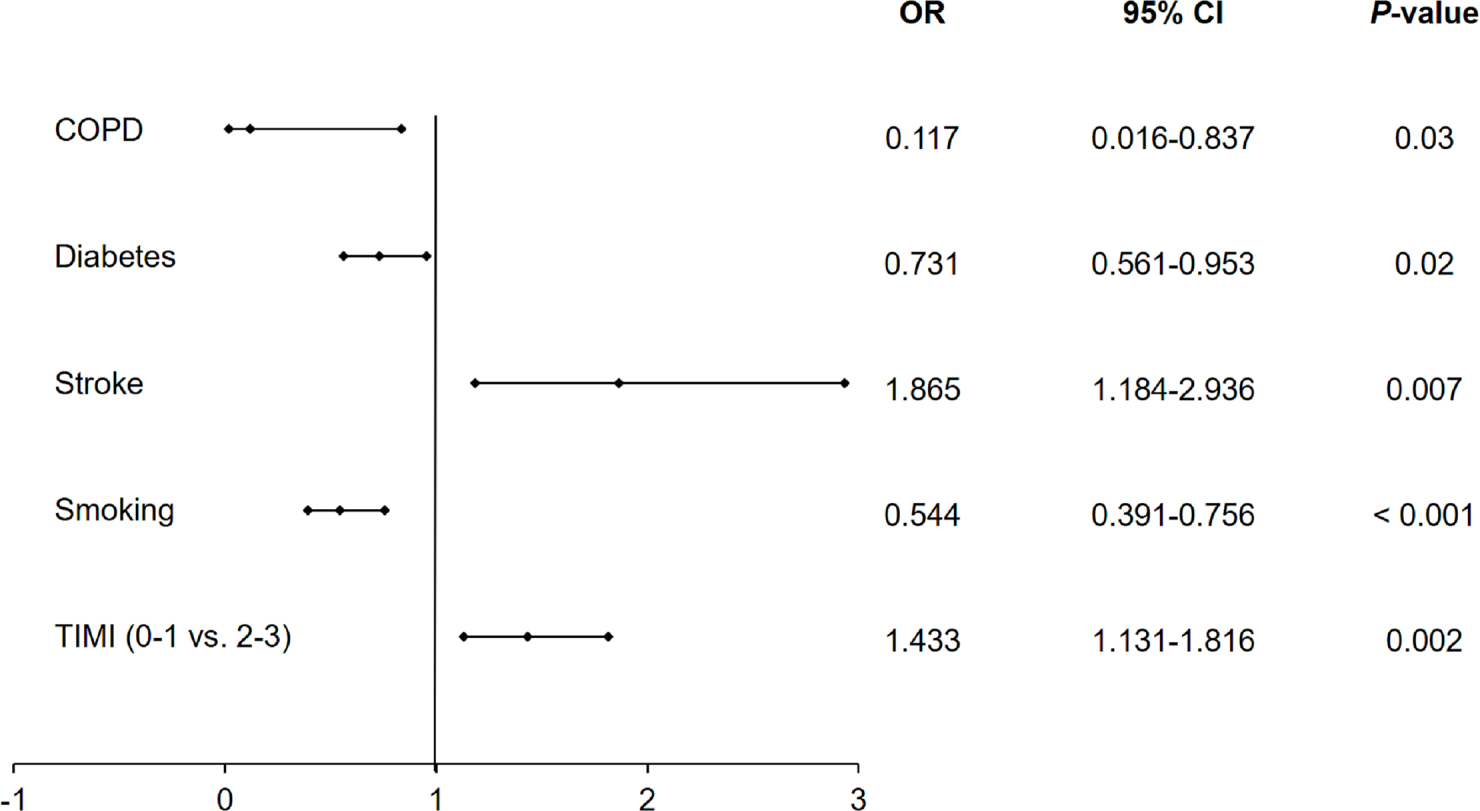
Predictors of allergic reactions during PCI assessed by multivariate analysis in the overall group of patients undergoing PCI. Abbreviations: CI, confidence Interval; COPD, chronic obstructive pulmonary disease; OR, odds ratio; TIMI, thrombolysis in myocardial infarction.

**Fig 4:**
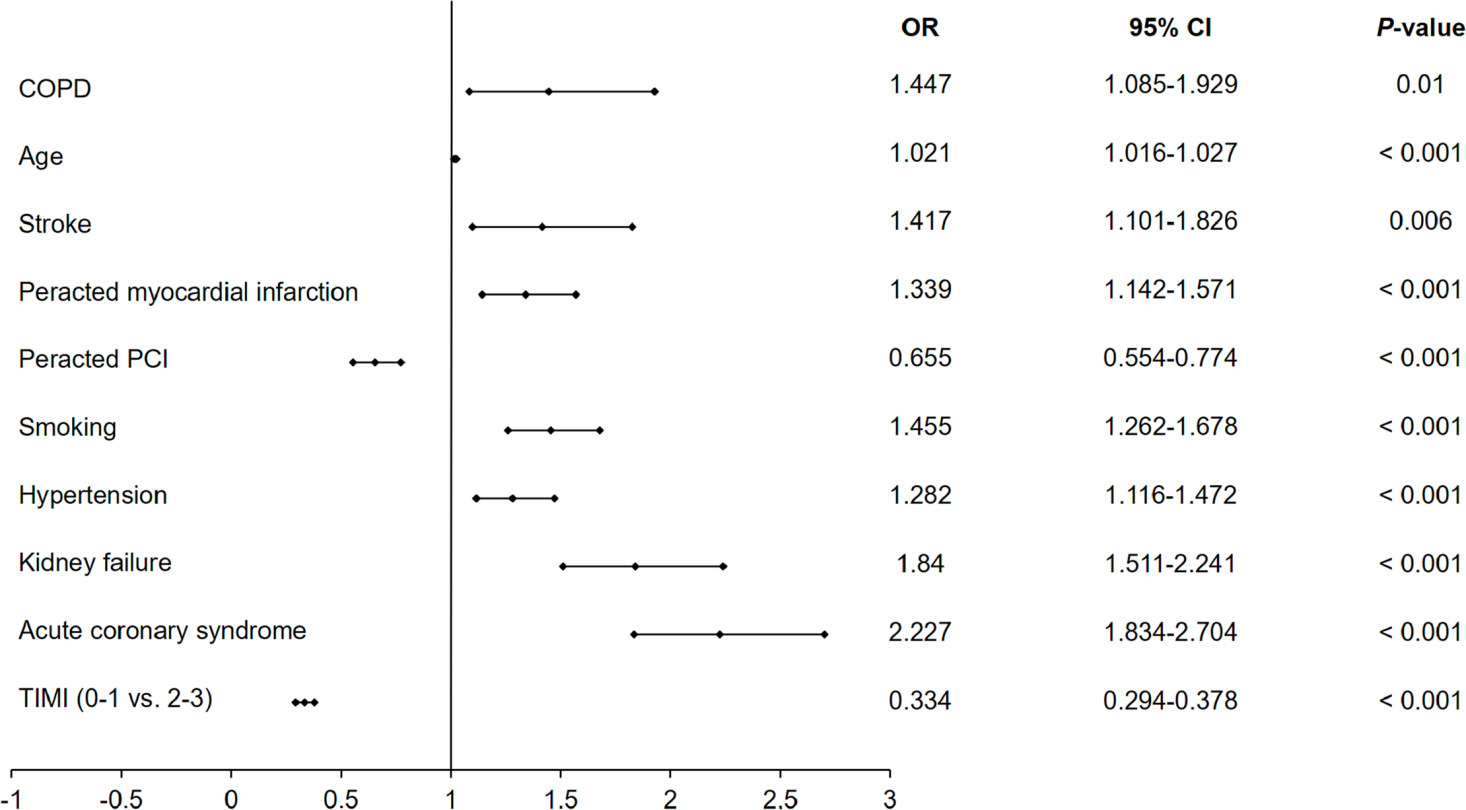
Predictors of no-reflow complications during PCI assessed by multivariate analysis in the overall group of patients undergoing PCI. Abbreviations: CI, Confidence Interval; COPD, chronic obstructive pulmonary diseases; OR, odds ratio; PCI, percutaneous coronary intervention; TIMI, thrombolysis in myocardial infarction.

Among several independent predictors of all periprocedural complication rates estimated by multivariate analysis in the overall group of patients undergoing PCIs, COPD was not confirmed to be an independent predictor (Fig 2). Using multivariate analysis, we displayed that hypertension and preserved blood flow before PCI are predictors of lower periprocedural complication rates, while increased age, male gender, diabetes, previous stroke, previous MI, psoriasis, chronic kidney disease and ACS were predictors of increased periprocedural complication rates (Fig 2). We also noticed that smoking, COPD and diabetes were among the independent negative predictors of periprocedural allergic reactions assessed by multivariate analysis, while stroke and preserved blood flow assessed by TIMI score before PCI were among factors increasing the probability of periprocedural allergic reactions (Fig 3). Moreover, COPD alongside with age, stroke, MI, smoking, hypertension, chronic kidney disease and ACSs were independent positive predictors of periprocedural no-reflows, while previous PCI and baseline TIMI 2–3 grade flow decreased the probability of no-reflow (Fig 4).

## Discussion

The main findings of the current study are that overall complication rates in patients with COPD are higher as compared to patients without COPD. Among all periprocedural complications, the rate of cardiac arrests, MIs and no-reflows was higher in patients from the COPD compared to the non-COPD group. Multivariate analysis did not confirm COPD as an independent predictor of periprocedural complications in the overall group of patients undergoing PCIs. Age, gender, diabetes, stroke, peracted MI, psoriasis, hypertension, kidney failure, ACS and TIMI flow before PCI were among the independent predictors of periprocedural complications confirmed by multivariate analysis. The third finding of the current study is that COPD and smoking along with diabetes were found to be a predictor of decreased periprocedural allergic reaction rate in patients undergoing PCI. The fourth significant finding of the presented study is that COPD and smoking were predictors of increased no-reflows in patients undergoing PCIs.

In the present study, the rate of periprocedural complications was comparable to the results published by other authors [5]. While our estimations indicate that the rate of periprocedural complications was significantly higher in the patients with rather than without COPD, the study published by Enriquez et al. reported a different proportion [5]. They estimated the rate of periprocedural complications in patients from the non-COPD group at 4.2%, which was significantly higher compared to the COPD group (2.6%; p = 0.004). In previously published studies, in-hospital mortality rate in the overall population ranges between 2.2%-2.83% and was significantly higher compared to those patients from the non-COPD groups [5,11]. Those death rates were higher compared to our results. However, we included only periprocedural deaths. Our analysis did not cover all hospital stays, which significantly underestimates the death rate. Previously published studies revealed higher rates of deaths in patients with COPD in the overall group of patients undergoing PCI and in patients with acute MI [5,12]. However, the study published by Sung et al. revealed a lower rate of in-hospital deaths in patients with COPD and STEMI undergoing primary PCI compared to non-COPD individuals. Furthermore they noticed an increased rate of recurrent MIs in the COPD group as compared to the non-COPD group (4.8% vs. 1.3%; p = 0.01) [13]. Although, their population of patients included more patients with MVD (59.7% vs. 34.4%) and baseline blood flow expressed as TIMI grade was 0–1 before PCI (80.6% vs. 30.3%) [13].

In the current study, we revealed a higher rate of periprocedural CAs, MIs and no-reflows in patients from the COPD group. The study published by Zhang et al. demonstrated an increased rate of in-hospital MIs in patients with rather than without COPD (16.3% vs. 4.6%) [14]. The study was conducted among a group of 2,362 patients undergoing PCI. Among them, 233 (9.8%) of patients were diagnosed with COPD. Since the cardiac mortality rate was zero in both groups, no differences were found between them. The important fact is that the authors did not mention any information about the distribution of CAD clinical presentation before PCI or the type of coronary atherosclerosis. Nevertheless, they also showed that the incidence of heart failure was greater in the COPD group as compared to the non-COPD group (22% vs. 9.2%) [14]. The increased rate of periprocedural MIs in patients with COPD as compared to the non-COPD group could be explained by more complex and disseminated coronary atherosclerosis as well as the difference in clinical presentation resulting from it. Whereas the increased rate of no-reflows and CAs could undoubtedly be related to the prothrombotic state attributed to COPD patients and the impeded return of blood flow through the vessel in STEMI patients related to it [15,16]. Our study confirmed that STEMI patients had a high rate of no-reflows (2.8%), CAs (2.6%), and the overall periprocedural complication rate was also high (6.2%) compared to other clinical presentations of CAD. Moreover, the relationship between no-reflows and the COPD as a prothrombotic state have been more widely discussed in two previously published manuscripts [17,18]. Furthermore, according to our knowledge, this is the first study verifying that among several confirmed predictors, smoking and COPD are independent risk factors of a higher incidence of no-reflows in patients undergoing PCI.

In contrast to our results, research conducted by other authors revealed that patients with COPD were more likely to experience major entry site complications than patients without COPD [16,19]. The leading entry site periprocedural complication was higher rate of bleeding that required transfusion in COPD patients. The study published by Enriquez et al. revealed that the rate of periprocedural access site bleeding requiring transfusion in patients with COPD was estimated at 2.3%, while in patients without COPD, it was at 1.4% (p = 0.03) [5]. We noted that puncture site bleeding rate was higher in patients with COPD compared to non-COPD patients but without statistical significance (0.2% vs. 0.15; p = 0.06). It was attributed mainly to age, gender and therapy implementing warfarin [16,19]. However, most studies maintain the thesis that the etiology of increased risk of periprocedural entry side bleeding is multifactorial [5].

Another worthy finding of the current study is that the rate of allergic reactions was higher in the COPD group as compared to the non-COPD group. Similarly, Mukherjee et al. observed a lower incidence of COPD in patients with respiratory allergy [20]. They noticed that among 550 participants, 18.9% of patients from the non-allergic population suffered from COPD, while 7.7% of allergic subjects had COPD [20]. Based on this, it could be concluded that patients with COPD and smokers present restrained allergic reactions. According to our knowledge, the present study is the first study which demonstrates that COPD and smoking are independent predictors of decreased periprocedural allergic reaction rate in the overall group of patients undergoing PCIs.

## Limitations

The ORPKI database does not specifically define particular periprocedural complications and is based on the operators’ discretion. Similar concerns could be applied to periprocedural complications and their definitions usually remain in the hands of operators. Additionally, assignment to the COPD and non-COPD groups was based on previous diagnosis and/or typical treatment while no data on spirometry test results were collected. This could bring some false positive or false negative diagnosis of COPD and bias related to this issue. However this is a typical problem for the national registry. From the other side it presents the real life and the real frequency of COPD diagnosis confirmed in spirometry is close to the presented in the study. Patients with exacerbated COPD at admission could be treated in some cases with systemic glucocorticoids, which might modify the extent and frequency of procedural-related allergic reactions.

## Conclusions

In the patients undergoing PCI, the periprocedural complication rate is higher in those from the COPD group than the non-COPD group. Cardiac arrests, myocardial infarctions and no-reflows may be found among the periprocedural complications with the largest contribution. COPD was not an independent predictor of periprocedural complications in the overall group of patients undergoing PCI, while COPD and smoking were found to be independent predictors of decreased allergic reaction periprocedural rate and increased rate of periprocedural no-reflows.
